# Impact of Mass Media on the General Population's Knowledge and Attitudes Toward Rheumatoid Arthritis in Qassim, Saudi Arabia

**DOI:** 10.7759/cureus.31079

**Published:** 2022-11-04

**Authors:** Mousa N Alrashdi, Sami M Alrasheedi, Ahmad Alkhdairi, Khalid O Almutairi, Mohammad A Almutairi, Abdullah F Alharbi, Asrar A Almutairi, Abdullah M Alsubaie

**Affiliations:** 1 Department of Medicine, Unaizah College of Medicine and Medical Sciences, Qassim University, Unaizah, SAU; 2 Department of Family Medicine, Qassim Ministry of Health, Unaizah, SAU; 3 Department of Medicine, King Fahad Medical City, Riyadh, SAU

**Keywords:** qassim, saudi arabia, rheumatoid arthritis, mass media, attitudes, knowledge

## Abstract

Objectives

This study aimed to assess the general population's knowledge of rheumatoid arthritis (RA), its health consequences, and the impact of mass media on their knowledge and attitudes in Qassim, Saudi Arabia.

Methods

A cross-sectional study was used through an online questionnaire to determine the general characteristics of participants and the influence of mass media on their knowledge, attitudes, and fears toward RA. An online pretested and standardized questionnaire was distributed through social media using combined convenience and snowball sampling that targeted the general population residing in Qassim, Saudi Arabia, between 10 January and 11 March 2022. Frequencies and percentages were employed as descriptive statistics. To examine the statistical differences in knowledge scores between various sociodemographic factors, the student’s t-test and analysis of variance were used.

Results

A total of 419 participants were included. About 20.3% of the participants were unaware of RA. Mean knowledge showed very low scores of 2.5 ± 2.24 out of 15. There were no significant differences in knowledge when it was compared with different sociodemographic variables, except for marital status and monthly income. There was a positive attitude toward the seriousness of RA. The correlation of knowledge scores with attitude and fear scores showed a poor or negligible correlation (rho= 0.130, p=0.008), whereas, with media influence, there was a low positive correlation (rho= 0.347 p<0.001).

Conclusion

This study found that even though our sample population had acceptable views concerning the significance of rheumatoid arthritis, their understanding of the condition was lacking. To promote knowledge of RA and its unfavorable health impact on affected individuals, public awareness initiatives with substantial media influence should be implemented.

## Introduction

Rheumatoid arthritis (RA) is a systemic inflammatory disorder that mainly affects the small joints, and it is the most common form of inflammatory arthritis that leads to disability [1]. It is difficult to determine the actual prevalence of RA in the Saudi population. In fact, the only study that looked at the frequency of RA in Saudi Arabia (SA) was published in 1998, which focused only on the Qassim region of SA and showed an estimated prevalence of 2.2 per thousand people [2]. Women were affected more than men, and the prevalence of the disease increased with multiple risk factors, including age [2]. The etiology of RA remains poorly understood despite advances in diagnostic and therapeutic measures [3]. The clinical presentation of RA varies, but an insidious onset of pain with symmetric swelling of small joints is the most frequent finding [4]. Although RA typically presents as symptoms associated with joint arthritis, manifestations involving other organs are also common and may precede the joint symptoms. Examples of extraarticular manifestations include interstitial lung disease (ILD), pleural effusions, pericarditis, and bronchiectasis [5].

The significance of early RA diagnosis stems from the fact that it has been linked to a reduced rate of joint destruction and a greater potential of disease-modifying anti-rheumatic medication (DMARD)-free remission [6]. Patients with RA frequently fail to reach specialist clinics early in the disease's progression. This could be because they prefer to consult orthopedic experts first, rather than rheumatologists, for their concerns [7]. In SA, for example, a study found that almost half of patients consult orthopedic surgeons first, whereas only one-third see rheumatologists [8].

Although the observed time from onset of symptoms to seeking medical attention is a major impediment to RA diagnosis and therapy, breakthroughs in RA treatment and the early introduction of medications have considerably decreased all-cause death and long-term disability [7,9-11]. The time from symptoms onset to the initial visit to a consultant physician (Lag1) was reported in a recent Saudi systemic review as 6.2 months while the time to initiation of DMARD therapy (Lag2) was 30.2 months [7]. However, in another study conducted in Saudi Arabia, the authors found that lag1 was 6.2 months while the combined lag1,2 was 5.27 months [6]. Patient education is crucial to maintain or ameliorate their health or, in some circumstances, to help slow down disease progression [12]. Although public education initiatives utilizing the media, internet, pamphlets, and professional counseling have been shown to be beneficial in increasing public knowledge of some diseases, such a campaign for inflammatory arthritis, especially rheumatoid arthritis, has not been launched frequently in Saudi Arabia [13,14]. Two studies done in SA addressed the public knowledge of RA in two different cities. One of them showed good knowledge of RA symptoms but a lack of knowledge about the causes and complications of RA, while the other study showed that nearly half of the participants were not aware of RA. The contradictory findings of these research papers prompted us to evaluate the level of knowledge of RA, as well as the mass media's effect and the necessity for programs and initiatives to raise awareness about the condition.

## Materials and methods

A cross-sectional survey was conducted among residents of the Qassim region, Saudi Arabia, using a mixture of convenience and snowball sampling techniques. Participants who were above 18 years old, with no gender discrimination, were invited to participate after obtaining consent from them.

A pretested questionnaire was used to collect the data from the participants. There were two language versions of the questionnaire (Arabic and English), and participants were given the choice of selecting one of the versions of the questionnaire. Two experts proficient in both languages performed a back translation validation process. The reliability of the questionnaire was checked by using Cronbach's alpha and the interclass correlation coefficient.

The questionnaire was divided into four sections. Section A contained an anonymity and confidentiality declaration that explained the objective and advantages of the study. It also documented the sociodemographic features of the individuals (age, gender, marital status, educational qualification, monthly salary, and whether diagnosed with RA or not). Sections B and C contained questions that assessed knowledge and attitudes toward RA, respectively. Section D documented the influence of the media on participants' perceptions of RA. Ethical approval was obtained from the Research and Ethics Committee of Qassim University.

Data management and statistical analysis

The data collected from the online survey were downloaded and transferred to a Microsoft Excel sheet (Microsoft Corporation, Redmond, WA), and data cleaning was done before statistical analysis. The IBM Statistical Package for Social Sciences, Version 23 (IBM Corp., Armonk, NY) was used for statistical analysis. Categorical data were presented using appropriate tables, with frequencies and percentages. A normality test was performed for all the continuous variables before choosing the appropriate test of significance. Continuous variables that showed normality were compared between categorical variables using student's t-test and/or analysis of variance (ANOVA), whereas the Mann-Whitney U and/or the Kruskal-Wallis H tests were utilized for those that didn't show normality. The correlation of the continuous variables was done using the Pearson correlation coefficient (r). A p-value less than 0.05 was considered statistically significant.

## Results

We received a total of 478 responses, of which only participants who satisfied the eligibility criteria were included in our analysis. Thus, we included a total of 419 participants for the final analysis. The participants' sociodemographic characteristics showed that 41.8% were 18-30 years aged, 54.4% were females, 63.2% were married, 74% were graduates, 46.3% had a monthly salary, and 8.4% had been diagnosed with rheumatoid arthritis (RA) (Table 1).

**Table 1:** Sociodemographic characteristics of the participants

		N	%
Age	18 to 30 years	175	41.8
	31 to 40 years	115	27.4
	41 to 50	92	22.0
	More than 50	37	8.8
Gender	Male	191	45.6
	Female	228	54.4
Marital status	Single	143	34.1
	Married	265	63.2
	Divorced	8	1.9
	Widow	3	0.7
Educational qualification	No formal education	2	0.5
	Primary	2	0.5
	Secondary	69	16.5
	Middle	16	3.8
	Graduate	310	74.0
	Postgraduate	20	4.8
Monthly salary (SAR)	< 5000	194	46.3
	5000 to 10000	104	24.8
	11000- 20000	109	26.0
	>20000	12	2.9
Diagnosed with rheumatoid arthritis	Yes	35	8.4
	No	384	91.6

In our study, about 20.3% of the participants were not aware of RA. It was found that about 46.8% disagreed that RA is a contagious disease, and 28.6% agreed that women are more likely to get the disease. About 35.8% had the knowledge that RA doesn't only affect the elderly, and only 16.9% knew that smoking is a risk factor for the development of RA. The responses to the knowledge related to symptoms and complications of RA are given in Table 2.

**Table 2:** Knowledge items responses by the participants * Correct Answer

	Responses (N (%))		
	Correct	Wrong	I don't know
Is rheumatoid arthritis a contagious disease?	9 (2.1)	196 (46.8)*	214(51.1)
Women more likely to get rheumatoid arthritis?	120(28.6)*	31 (7.4)	268(64.0)
Rheumatoid arthritis only affects the elderly?	50(11.9)	150(35.8)*	219(52.3)
Is smoking one of the most important risk factors for rheumatoid arthritis?	71 (16.9)*	55 (13.1)	293 (69.9)
Rheumatoid arthritis is characterized by pain in the joints of the hands and stiffness in the morning for more than half an hour?	24 (5.7)	110 (26.3)	285 (68.0)
Rheumatoid arthritis is characterized by abdominal pain	21 (5.0)	118 (28.2) *	280 (66.8)
Rheumatoid arthritis is characterized by inflammation of the small joints such as the joints of the fingers and toes.	127 (30.3)*	28 (6.7)	264 (63.0)
Rheumatoid arthritis affects only the joints of the knees?	58 (13.8)	114 (27.2)*	247 (58.9)
Rheumatoid arthritis affects all joints in the body with no exceptions.	139 (33.2)*	33 (7.9)	247 (58.9)
Rheumatoid arthritis is characterized by an increase in pain with movement?	173 (41.3)	18 (4.3)	228 (54.4)
If not treated, rheumatoid arthritis may cause deformities in the affected joints, leading to loss of function in the absence of treatment?	162 (38.7)*	14 (3.3)	243 (58.0)
Rheumatoid arthritis patients often end up in a wheelchair	82 (19.6)	53 (12.6)*	284 (67.8)
Does rheumatoid arthritis affect body organs such as the heart and lungs?	82 (19.6)*	48 (11.5)	289 (69.0)
Is it better to have rheumatoid arthritis diagnosed and treated by an orthopedic specialist?	224 (53.5)	26 (6.2)*	169 (40.3)
There are medicines that relieve pain and prevent joint deformities, but do not treat the disease permanently?	198 (47.3)*	14 (3.3)	207 (49.4)

There was a total of 15 knowledge items in the questionnaire. The knowledge scores for each participant were calculated using the responses, where correct responses were given a score of 1, and wrong or unaware answers were given a score of 0. Thus, the maximum score one could score was 15, and the minimum was 0. Our analysis showed that the mean knowledge score in our study was 2.06 ± 2.05 (minimum= 0; maximum=9). We found that about 131 participants (31%) had given a total knowledge score of 0, which shows that the knowledge related to RA is poor. When we compared the knowledge scores between different sociodemographic characteristics, only the marital status and salary of the participants showed statistically significant differences. The mean knowledge scores of participants who were single (2.5 ± 2.24) and those who had a salary of fewer than 5000 riyals (2.37 ± 2.13) showed comparatively higher scores than their counterparts (p<0.05) (Table 3).

**Table 3:** Comparison of knowledge level with sociodemographic characteristics * p value <0.05 is considered statistically significant

		N	Mean	Standard deviation	P value*
Gender	Male	191	1.8848	2.08688	0.106
	Female	228	2.2105	2.01524	
Marital status	Single	143	2.5245	2.24183	0.003
	Married	265	1.8642	1.91001	
	Divorced	8	0.6250	1.40789	
	Widow	3	1.3333	2.30940	
Educational level	No formal education	2	0.5000	.70711	0.654
	Primary	2	2.5000	3.53553	
	Middle	16	2.3750	2.41868	
	Secondary	69	1.8986	1.94894	
	Graduate	310	2.1194	2.06887	
	Postgraduate	20	1.6000	1.84676	
Salary (SAR)	< 5000	194	2.3763	2.13475	0.022
	5000 to 10000	104	1.9135	1.89589	
	11000- 20000	109	1.7248	1.99941	
	>20000	12	1.3333	1.87487	
Diagnosed with RA	Yes	35	2.4000	2.26482	0.309
	No	384	2.0313	2.03213	

The responses of participants related to the influence of media on RA awareness are given in Table 4. It was found that media had a poor influence, where 74.7%, 79.7%, 86.2%, and 72.3% never read a comprehensive article in magazines or newspapers, watched a TV show, listened to a radio program, or read a scientific publication about RA, respectively. The mean score for media influence was found to be 1.45 ± 2.45, which showed that there was poor media influence related to RA on the participants (Table 4).

**Table 4:** Media influence on participants

	Responses (scale score)				Total mean score (SD)
	Never (0)	Sometimes (1)	Often (2)	Very often (3	
Read a comprehensive article in magazines or newspapers about RA during the past year	313 (74.7)	88 (21.0)	12 (2.9)	6 (1.4)	1.45 (2.45)
Watched a TV show about RA during the past year	334 (79.7)	62 (14.8)	17 (4.1)	6 (1.4)	
Listened to a radio program about RA during the past year	361 (86.2)	42 (10.0)	10 (2.4)	6 (1.4)	
Read a publication about RA	303 (72.3)	92 (22.0)	14 (3.3)	10 (2.4)	
Read a comprehensive article about rheumatoid arthritis in magazines or newspapers during the past year	317 (74.7)	88 (21)	12 (2.9)	6 (1.4)	

The attitudes and fear concerns related to RA were assessed, and it was found that 40.8% reported that they would get nervous if they heard about patients who got paralyzed due to RA, and 45.4% mentioned that they get worried when they came to read about adverse effects of a drug that RA patients take. It was reported by 45.3% that they would feel nervous if a friend told them that he/she had RA. More than half of the participants said that if they felt any pain in their neck or shoulder for a while, they would fear RA. The majority of the participants (64.7% agreed that they would visit a doctor if they felt soreness or stiffness in the body joints). It was reported by 76.6% of the participants that they would be concerned and visit a doctor if they experience one or more joint swelling. The mean scores of attitude and fear concerns were calculated and the means score was calculated, where a higher score indicates poor attitude and more fear. The mean score was found to be 21.01 ± 5.01, which means that the study participants had a poor attitude and more fear related to RA (Table 5).

**Table 5:** Attitude and other concerns about rheumatoid arthritis

	Responses [n(%)]					
	Strongly Agree (5)	Agree (4)	Neutral (3)	Disagree (2)	Strongly Disagree (1)	Total Mean Scores
When I see a TV show about patients paralyzed by arthritis, I get nervous.	71 (16.9)	100 (23.9)	145 (34.6)	69 (16.5)	34 (8.1)	21.01 (5.01)
I get worried when I read about the side effects of a drug that arthritis patients take.	72 (17.2)	118 (28.2)	138 (32.9)	67 (16.0)	24 (5.7)	
Would I feel nervous if a friend told me he had arthritis?	65 (15.5)	125 (29.8)	113 (27.0)	77 (18.4)	39 (9.3)	
If I felt pain in my neck and shoulder for a while and thought I had arthritis, I would be concerned	74 (17.7)	155 (37.0)	88 (21.0)	76 (18.1)	26 (6.2)	
If I frequently feel sore and stiff when I wake up, I will go to the doctor	140 33.4	131 31.3	82 19.6	49 11.7	17 4.1	
I think that swelling of one or more joints is cause for concern and will lead me to visit the doctor and do a complete examination.	151 (36.0)	170 (40.6)	57 (13.6)	27 (6.4)	14 (3.3)	

The correlation of knowledge scores with attitude and fear scores showed a poor or negligible correlation (rho= 0.130, p=0.008), whereas, with media influence, there was a low positive correlation (rho = 0.347, p<0.001) (Table 6).

**Table 6:** Correlations of knowledge with attitude and fear, and media influence scores

		Attitude and Fear Scores	Media Influence Scores
Knowledge score	Pearson correlation	0.130	0.347
	P value	0.008	0.000
	N	419	419

## Discussion

The purpose of this study was to discover how much the public knows about RA, its health consequences, and how the media influenced their knowledge of the disease. The outcomes of this study revealed that the participants' understanding of RA was lacking. In contrast, communities in Egypt, Iraq, Netherlands, and Portugal showed higher knowledge of the condition, with more than half of the research participants knowing of RA [15-18]. The analysis showed that the participants received a mean score of 2.06 out of 15 items regarding general knowledge of RA. For most of the knowledge items, the majority of the participants responded that they did not know about the statements mentioned. Nearly one-third of the participants agreed that RA is characterized by inflammation of the small joints such as the fingers and toes. Therefore, it is clear that participants in the current population do not have adequate awareness of the various presentations. One study in Saudi Arabia found similar results to ours, with respondents demonstrating poor knowledge of RA while another study found good knowledge of RA [13,14]. It is worth noting that in both trials, over half of the individuals were unaware of RA, whereas just one-fifth of our sample was unaware of the disease [13,14]. Furthermore, Hazzazi's study showed that 52% of participants accurately identified that RA is more frequent in women as compared to 28% in our study [13]. People who learn more about RA may acknowledge that patients are not to blame for the outset of their disease and can't change their disease trajectory. In general, there was a difference in studies outcomes regarding awareness and how to address the disease manifestations, both in terms of its potential fatality and the disabilities from it. In this study, the participants had good intentions of going to the doctor if they had rheumatic complaints. The knowledge of the participants correlated positively with the impact of the media. Participants who had good knowledge of the disease had more fear regarding the complications of the disease. However, the participants' knowledge didn't show a strong correlation with behavioral intentions, attitude, and fear about the seriousness of the consequences of RA.

Most of the participants have not used the media often enough to get information about RA. However, they showed a moderate willingness to receive more information about rheumatic diseases. Although individuals in different European nations (for example, the Netherlands) and the United States benefit more from group education which increases their understanding and attitude regarding the condition [19]. Additionally, more information on RA in the mainstream media will contribute to a more positive perception of the disease among the general public [17]. Through media coverage, rheumatic disorders could be brought to the attention of people who have never suffered from them. When society begins to recognize rheumatic diseases, patients will be more willing to come forward and share their experiences. In opposition to our results, it was reported in a previous study that people with lower educational qualifications seemed to have less knowledge of RA, and people with higher qualifications felt more vulnerable about getting the disease even though they didn't have higher intentions of visiting a doctor if they experienced rheumatic complaints [17]. The media's information regarding RA should be targeting young people, females, those with poor educational qualifications, positive family history of RA, previous joint injury, smokers, obesity, etc. Public awareness initiatives for lower back pain, for example, indicated that the campaign effectively raised public knowledge, even three years after the media campaigns ended [20]. According to previous studies, education is not one program but rather a strategy suited to each group, and providing educational content alone does not have a positive influence [21].

The study's primary purpose was to understand more about the local general population's awareness and attitude toward mass media in regard to RA and to discuss what could be required to persuade the general public to support research on this subject. As a result, the rather comprehensive evaluation of the scope of the problem provided by this study is concluding that knowledge of RA was not adequate and mass media influence was less than expected. In Saudi Arabia, there have never been any equivalent studies conducted, and thus it is difficult to compare our findings with previous efforts in this field. Nevertheless, if further improvement is to be done, the current findings indicate that targeted public education by using various media should be expanded to facilitate early recognition of RA patients and to reduce disability risks, which, in turn, leads to a positive impact on different facets of quality of life.

Some of the limitations of this research should be addressed before interpreting our findings. First, we used self-reported, online survey methods, and this might give the possibility of invalid answers. Due to the abovementioned nature of response collection, social desirability bias and response bias are also possible. Another issue with self-reported questionnaires is the lack of clarity in the questions, which increases the likelihood of conflicting interpretations of queries. Second, our sample size was small, and future studies should examine whether these findings are replicated with a larger sample. Finally, evidence suggests that implicit attitudes may be more accurate than explicit self-reported attitudes in predicting adherence to actual attitudes [22]. Another drawback of this study is that we did not utilize an objective measure for actual (rather than self-reported) attitudes and fear toward RA and media influence.

## Conclusions

This study found that even though our sample population had acceptable views concerning the significance of rheumatoid arthritis, their understanding of the condition was lacking. The media's impact has been found to have relatively favorable connections with participants' understanding of rheumatoid arthritis. To promote awareness about the gravity of this illness and its influence on the quality of life of those affected, public awareness programs about the risk factors, diagnosis, and management of rheumatoid arthritis should be launched.
